# Glycerol Valorization—The Role of Biochar Catalysts

**DOI:** 10.3390/molecules27175634

**Published:** 2022-09-01

**Authors:** Ana R. P. Gonçalves, Ana P. C. Ribeiro, Sofia Orišková, Luísa M. D. R. S. Martins, Ana F. Cristino, Rui Galhano dos Santos

**Affiliations:** 1 CERENA, Instituto Superior Técnico, Universidade de Lisboa, Av. Rovisco Pais, 1049-001 Lisboa, Portugal; 2 Centro de Química Estrutural, Institute of Molecular Sciences and Departamento de Engenharia Química, Instituto Superior Técnico, Universidade de Lisboa, Av. Rovisco Pais, 1049-001 Lisboa, Portugal; 3 Centro de Química Estrutural, Institute of Molecular Sciences and Departamento de Química e Bioquímica, Faculdade de Ciências, Universidade de Lisboa, Campo Grande, 1749-016 Lisboa, Portugal

**Keywords:** glycerol, catalysis, catalyst, biochar, biomass

## Abstract

The conversion of renewable feedstocks into new added-value products is a current hot topic that includes the biodiesel industry. When converting vegetable oils into biodiesel, approximately 10% of glycerol byproduct is produced. Glycerol can be envisaged as a chemical platform due to its chemical versatility, as a scaffold or building block, in producing a wide range of added-value chemicals. Thus, the development of sustainable routes to obtain glycerol-based products is crucial and urgent. This certainly encompasses the use of raw carbonaceous materials from biomass as heterogeneous acid catalysts. Moreover, the integration of surface functional groups, such as sulfonic acid, in carbon-based solid materials, makes them low cost, exhibiting high catalytic activity with concomitant stability. This review summarizes the work developed by the scientific community, during the last 10 years, on the use of biochar catalysts for glycerol transformation.

## 1. Introduction

The search for new environmentally friendly fuels has gained considerable interest due to petroleum-based fuels’ adverse environmental impact and its expected depletion. In addition, the United Nations conference on climate change, COP21, brought public awareness to the requirements needed to mitigate climate change, and 17 Sustainable Development Goals (SDGs) were established [1] within the 2030 Agenda for Sustainable Development. The use of greener fuels to address such envisaged goals is considered as one of the most promising solutions, unlocking a competitive, low carbon, and energy efficient future [2].

By using wastes as feedstock to produce fuels, a new sustainable alternative to traditional petrochemical feedstock processes (avoiding uncertain availability and environmental concerns) can be attained [3]. However, the chemical conversion of biomass resources and the utilization of all by products is one of the most important challenges nowadays. The impact in society and the level of awareness that already exists inside and outside the scientific community makes the challenge of improving conversion of biomass to commodities a hot topic.

In converting vegetable oils into biodiesel, approximately 10% of glycerol (1,2,3-propanetriol) is produced as a byproduct [4,5], representing the main bottleneck in the biodiesel production chain. It is estimated that glycerol production will reach approximately 3.2 billion liters by 2025 [6,7]. The market value of pure glycerol is US$0.27–0.41 per pound; however, crude glycerol with 80% purity is as low as US$0.04–0.09 per pound [8].

The generation of massive amounts of this polyol has given rise to new challenges to overcome the surplus by its sustainable use.

**Figure 1:**
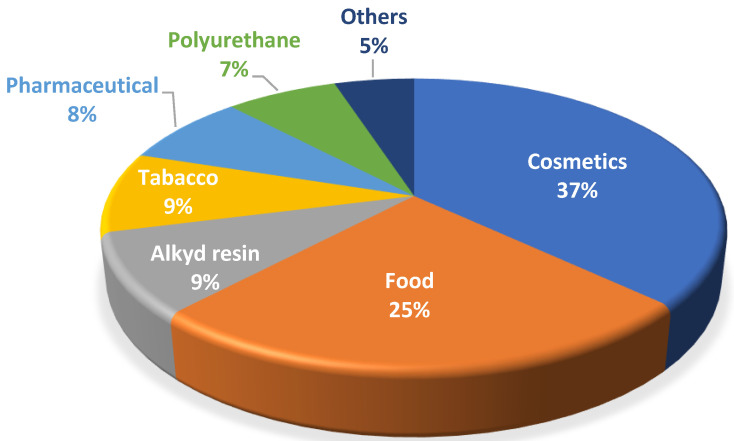
Distribution of the main glycerol applications [9].

Although there is a wide range for glycerol applications (Figure 1) [9], they are limited by its purity, affecting its physical, chemical, and biological properties. Applications in cosmetics, paints, drugs, paper, textiles, explosives, and as one of the key platform compounds to produce biopolymers and other high-value chemicals [10,11], makes the chemical transformation of glycerol highly relevant for crude glycerol valorization.

In the chemicals market, the production of biobased products has become a priority for the industries of plastics, solvents, lubricants, and surfactants. The forecast period of 2019–2027 [12] expects an increase at a compound annual growth rate (CAGR) of 16.67%.

**Figure 2:**
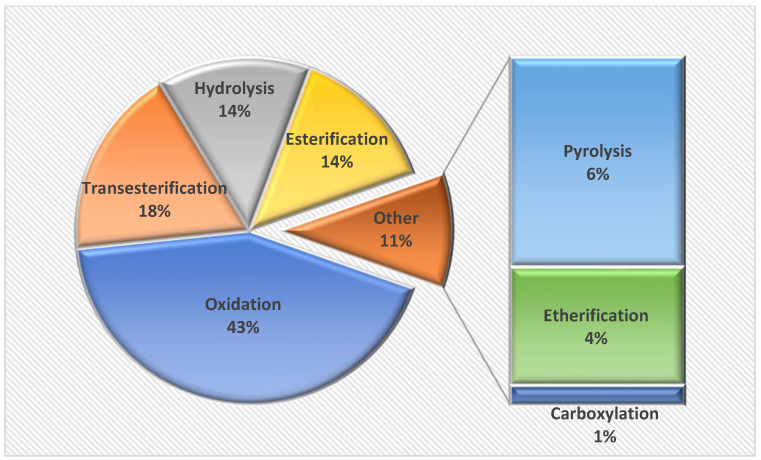
Processes for conversion of glycerol into added-value chemicals according to the number of articles published on the subject in the past eight years.

Four main factors showing glycerol’s great potential are [13]: (i) its availability, (ii) its low commercial value, (iii) being a renewable raw material, and (iv) providing economically viable alternatives for the biodiesel industry. Thus, recently, glycerol has undergone a series of catalytic conversion processes that could be useful in producing new added-value products. These reactions led to further investigation on developing and improving ways to obtain such products from glycerol with industrial application. More than thirty thousand scientific publications addressed glycerol in the last ten years. The catalytic glycerol valorization has gained attention [14], in particular, its oxidation, transesterification, esterification, and hydrolysis (Figure 2).

However, when such type of reactions uses biobased catalysts derived from biomass or waste materials, the number of scientific publications drops significantly, where the most addressed processes for the catalytic conversion of glycerol are transesterification, esterification, and etherification. Herein, the latest studies on the use of biobased catalysts for the above glycerol reactions, are presented and discussed.

## 2. Glycerol Conversion Using Biochar Catalysts

The real challenge for researchers and industries is to improve both catalysts and the technologies needed to convert feedstocks into added-value commodities [15]. 

Undoubtedly, catalysts are the workhorses of these processes. Due to their high efficiency, the most usual catalysts for glycerol conversion are homogeneous acids. This type of catalysts has several disadvantages, including the high cost of separating the catalyst from the reaction mixture, the large volume of waste products and environmental pollution. Heterogeneous catalysis, preferred by industry, is nonetheless far from perfect: high temperatures lead to energetic overconsumption, low selectivity raises environmental concerns, and deactivation can render processes complex, unecological, and uneconomical. 

Thus, environmental and economically superior catalytic processes exhibiting reduced reaction times, increased yield, and selectivity are essential to achieve progress in their sustainability. In this respect, the development of low-cost and high-active based catalysts from waste materials, rather than chemical reagents, has drawn increased attention in transesterification, esterification, etherification, and interesterification reactions, as presented below.

Although the heterogeneous catalysts have certain advantages it is important to remind ourselves that when assessing the environmental issues in the functionalization of carbonaceous supports, one uses the same homogeneous acids that makes the process environmentally challenging.

### 2.1. Transesterification

The interest in cyclic organic carbonates as potential commodity chemicals has grown in the past years. Cyclic carbonates, in particular, five-membered ones, are used as excellent (high boiling point) polar aprotic solvents [16,17], industrial lubricants, electrolytes for lithium-ion batteries [18], synthetic building blocks for polymeric materials [19,20], monomers to produce polycarbonates [21,22] as well as other fine chemicals [23,24]. Their features, such as high boiling and flashpoints, low toxicities, odor levels and evaporation rates, biodegradability, and solubility in a large range of solvents, make them a target of interest.

**Scheme 1:**
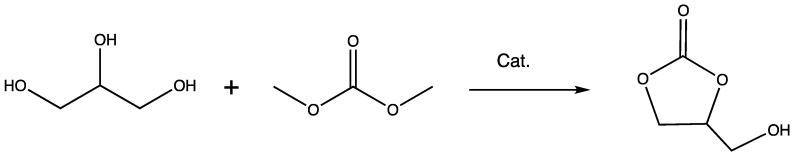
Catalytic transesterification of glycerol with dimethyl carbonate (DMC) into glycerol carbonate (GC).

Synthetized from the transesterification of glycerol with the non-toxic dimethyl carbonate (DMC), in the presence of a catalyst, glycerol carbonate (GC) (Scheme 1) is a good example that occupies a protuberant place in applications, such as polymer chemistry [25,26], or as fuel additive [27,28,29,30], solvent [31], or chemical intermediate [32]. Either choosing an acid or basic catalyst, homogeneous or heterogeneous, high reaction rate and selectivity are possible to achieve. 

Due to their abundance and high catalytic activity, heterogeneous calcium-based catalysts have been extensively investigated for glycerol transesterification [33]. However, due to hygroscopicity and instability, alternatives have emerged. The reusability of such catalysts in successive experimental cycles is another difficulty that has been pointed out, namely the serious deactivation due to severe leaching.

Catalytic transesterification of glycerol with DMC to GC over functionalized wastes as catalysts has received limited attention in the past years, with more emphasis on heterogeneous catalysts, such as biochar. Their relatively high surface area, tailored textural characteristics, and low cost make them excellent for transesterification reactions. These advantages have encouraged Changmai and co-workers [34] to use *musa acuminata* peel ash (MAPA) as a catalyst for the transesterification of glycerol under microwave irradiation. With a catalyst loading of 6 wt.%, a 1:2 molar ratio of glycerol:DMC (G:DMC), at 75 °C for 15 min, the authors attained a high conversion (99%) with excellent selectivity (99.5%). The use of conventional heating instead of the microwave-assisted reaction led to only 18% of glycerol conversion with 98.5% selectivity. Thus, once again microwave irradiation has proved to be effective in saving energy as reactants are heated by transferring electromagnetic radiation (low frequency oscillating electric and magnetic fields) directly into the molecules, while leaving the reactor unheated [35].

Changmai et al. [34], have further investigated the reusability of MAPA catalyst in up to 6 successive catalytic cycles, achieving 91% of glycerol conversion into GC after the 6th cycle. Despite the slight leaching of active sites detected in each catalytic cycle, no significant loss of catalytic activity was observed due to excellent stability (confirmed by TEM and EDX analyses). In contrast with the high calcination temperatures associated to the use of other solid catalysts from natural waste, these authors have developed a more energy-efficient protocol for the transesterification of glycerol with DMC to GC. 

**Scheme 2:**
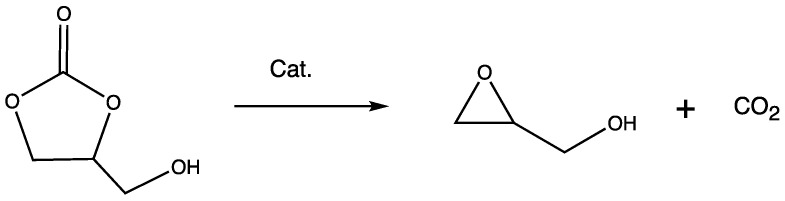
Catalytic decarboxylation of glycerol carbonate (GC) to glycidol.

Shikhaliyev and co-workers [36] employed fishmeal, producing a biochar to act as a catalyst in the transesterification of glycerol with DMC (Scheme 1). Fishmeal biochar carbonized at temperatures between 550–750 °C was used, and the most suitable temperatures based on glycerol carbonate yield was chosen for the transesterification reaction. Factors, such as reaction temperature, dosage of biochar, G:DMC ratio, and reaction time were investigated. GC yield and conversion increased steadily from 70 to 85 °C. The limit for the temperature to control the reaction in the liquid phase was 85 °C since their apparatus was not a high-pressured autoclave reactor that could contain the evaporation (DMC boils at 90 °C [37]). However, it would be interesting to investigate higher temperatures. The most suitable conditions were set as 85 °C, 2 wt.% dosage of biochar carbonized at 650 °C, G:DMC molar ratio of 1:2 under 60 min, leading to 99.5% yield of glycerol carbonate with a complete conversion of glycerol. The authors also found that GC yield and glycerol conversion marginally increased by varying the initial catalyst concentration from 1 to 2 wt.%, leaving some doubts about the need for doubling the catalyst concentration. As for the reaction time, it was found that for reaction times higher than 60 min, GC decarboxylation would occur, leading to an increasing amount of glycidol (2,3-epoxy-1-propanol, Scheme 2), and the released CO_2_ competed for available basic sites on the biochar. Methanol was added after each catalyst reuse without pre-treatment to maintain the high catalytic activity. This cost-effective and less energy-intensive method maintained a high yield of GC (93%) after five consecutive catalytic cycles. However, significant changes in mineral elements of biochar structure were detected, with evidence of a decreased carbon content and consequent increase in oxygen and calcium contents. A decrease in alkali metals (Na and K) and nitrogen was also observed. These changes justified the 0.8% difference in basicity after five consecutive catalytic cycles. 

In another work by the same group [38], potassium fluoride (KF) was added to enhance the biochar’s basicity. As in previous studies, methanol additions were also employed to respond to deactivation, improving GC yield after reuses. Two types of biochar were made: from cow dung (CDB) and fish scale (FSB) biowastes, both with additions of KF. These wastes were carbonized at various temperatures (500 °C to 750 °C), and GC yield was evaluated in search for the optimal temperature. For CDB, the highest GC yield (97.1%) was achieved at 600 °C, while for the unmodified FSB, the highest GC yield was only half of that value (48.6%) at 650 °C (FSB-650). The addition of the KF for this type of biochar promoted reaction conversion and the GC yield. Additions from 1 to 3 wt.% were studied, affording a maximum GC yield of 99.6% at 3 wt.% KF loading on FSB-650, with total conversion of glycerol. KF loads on FSB-650 above 3 wt.% led to the production of glycidol by decarboxylation of generated GC due to the strong alkaline properties of KF. Different optimal reaction times were obtained for both types of biochar, 60 min for 3% KF/FSB-650, and 90 min for CDB catalysts, being, according to the authors [38], amongst the least reported reaction time in this area of study. 

Unlike in previous studies, the biochar displayed relatively high catalytic activity at equimolar DMC/glycerol molar ratio. Despite the evidence that deactivation is inevitable with reusability, smaller GC yields were obtained after fewer cycles, reaching 81.3% and 82.6% for 3%KF/FSB-650 and CDB-600 catalysts, respectively, after four cycles. Ca, K, and Na content of the CBD-600 stayed nearly stable, but all the mentioned elements leached significantly on 3%KF/FSB-650.

Although all authors state that the catalyst they are using is cost-effective, these studies often lack information regarding their preparation costs. Being able to transform waste into a catalyst with high catalytic activity does not mean the catalyst is by itself low-cost, especially when there are pre-treatments using expensive chemicals. 

In the work of Wang and co-workers [39], a temperature of 500 °C was used to prepare the corncob residue catalyst CCR-500 that showed relatively high basicity and good catalytic activity. The structural characterization indicated that this catalyst was composed of carbon and some alkaline mineral salts. The optimal conditions for glycerol conversion were set at 80 °C for 90 min, with a catalyst amount of 3 wt.% and a G:DMC molar ratio of 1:3. As stated by the authors, catalysts are usually evaluated from two perspectives: their performance, including their catalytic activity and reusability, and their preparation cost. The latter can be divided in two categories: high-cost and low-cost catalysts. It is important to understand the difference between these two categories. The so-called high-cost catalysts often use expensive chemical reagents during preparation, and thus their relatively complicated preparation procedures do not meet the requirements for industrial applications, besides their acceptable performance. The low-cost catalysts are the ones prepared by using waste materials. These are often successfully obtained by more straightforward preparation procedures, thus translated into lower preparation costs.

In Table 1, and according to the findings by Wang et al. [39], a comparison is made for three catalysts, the high-cost [NiFe_2_O_4_@(CaO-La_2_O_3_)], the low-cost K-zeolite, and the biobased CCR-500. 

All catalysts present similar glycerol conversion yields. However, after reuse, the biobased catalyst differs from the others, with a decrease of 20%, after only four successive catalytic cycles. Nevertheless, the hypothesis of using a waste that represents up to 17% of the total weight of the overall corn production, mainly discarded or burned, is an advantage to consider. Another important note is that, even with this deactivation, biobased catalysts can be reactivated with additives prior to their reuse.

Table 2 presents a performance comparison of all above biochar catalysts made from different waste feedstocks for glycerol transesterification. 

With almost total glycerol conversion in all cases, the above biochar catalysts are able to operate at mild conditions and provide excellent selectivity to the desired glycerol carbonate.

**Figure 3:**
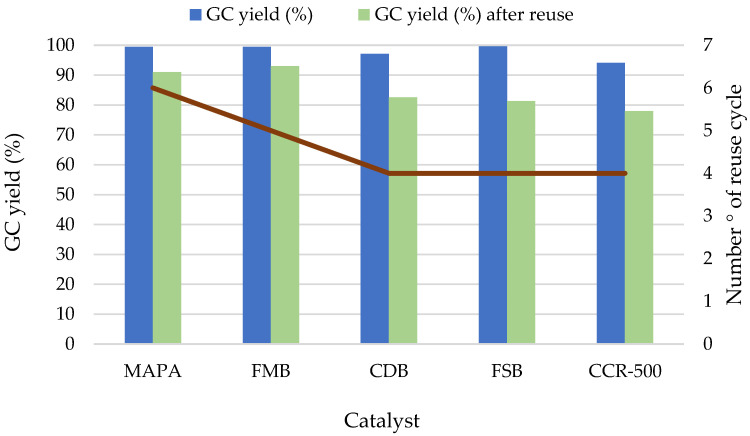
Comparison of GC yield (%) obtained before and after reutilization for several types of biobased catalysts made from different wastes.

Regarding biochar catalyst’s reusability in transesterification reactions, Figure 3 compares GC yield (%) obtained before and after reutilization. Even with variations in their reusability, all biochar catalysts could be reused from 4 to 6 cycles. A GC yield decrease up to 20% could be detected due to deactivation, which is comparable to other non-biobased catalysts. 

### 2.2. Esterification

Esters of glycerol, acetins, have a wide application in pharmaceuticals, cosmetics, food and fuel additives, and plasticizers, and they act as precursors for other chemical commodities [42].

**Scheme 3:**
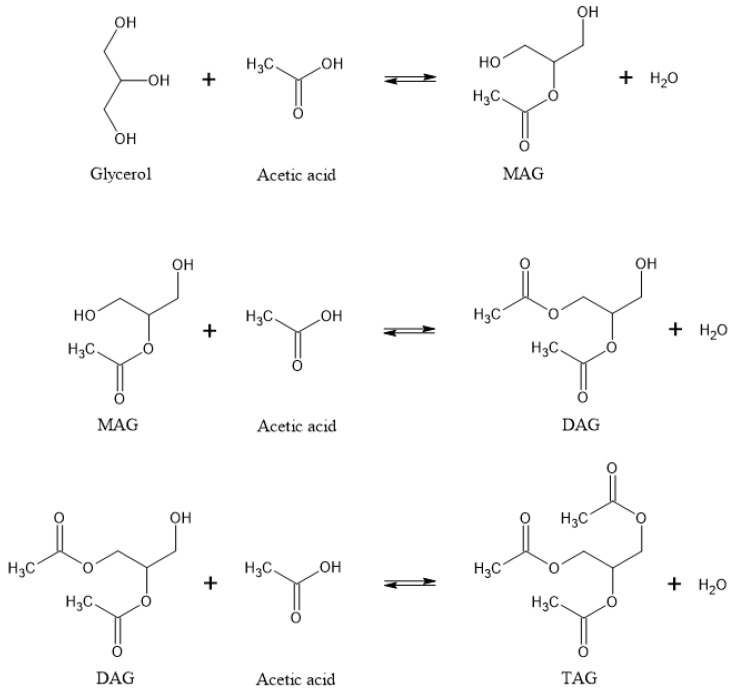
Glycerol acetylation with acetic acid to mono-, di- and triacetins (MAG, DAG, and TAG, respectively; adapted from ref. [46]).

In recent years, a large variety of solid acid catalysts employed in the acetylation of glycerol with acetic acid (AA) were reported [43,44,45]. This acetylation process includes a series of consecutive steps (Scheme 3), occurring in the presence of a catalyst, producing mono-, di- and triacetins: MAG, DAG, and TAG, respectively. In the first step, the reaction yields MAG and water. Further acetylation progresses, as the reaction time increases, leads to DAG from MAG and TAG from DAG, having water as a byproduct. The selectivity of each acetin increases at the expense of decreasing the one formed prior to it.

The selectivity to MAG, DAG, and TAG depends not only on the catalyst features, such as surface acidity, pore structure and stability, but also on esterification parameters, such as temperature, reaction time, glycerol to acetic acid molar ratio (G:AA), and catalyst amount. 

**Figure 4:**
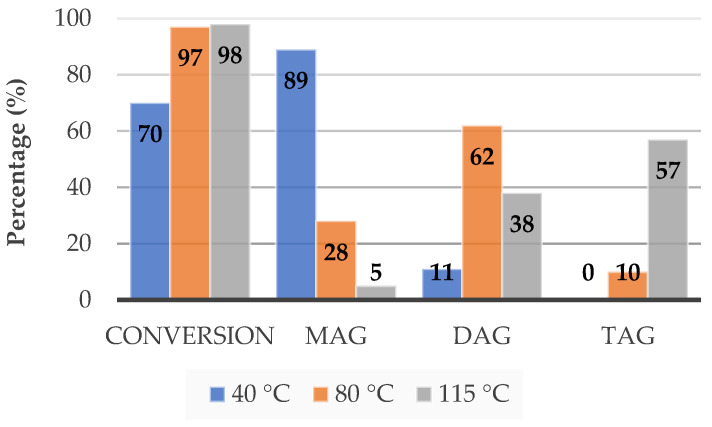
Effect of temperature on conversion and selectivity for the 1:9 G:AA molar ratio conditions.

In the work of Calle et al. [47], sulphonated hydrothermal carbon (SHTC) catalysts were obtained from D-glucose, by mild hydrothermal carbonization (19 h at 195 °C) and subsequent sulfonation with sulfuric acid (15 h at 150 °C) as nanospheres smaller than some of the reported analogous materials (300–400 nm diameter). The activity of SHTC catalysts in the acetylation of glycerol with acetic acid was investigated. Two reaction parameters, temperature (bath temperature 40, 80 and 115 °C), and G/AA molar ratio (1:3, 1:6, and 1:9) were evaluated, having conversion of glycerol and selectivity toward MAG, DAG, and TAG as response factors. These reactions were compared with homologous ones in the absence of the SHTC catalyst but only at two temperatures: 40 °C and 115 °C. Other parameters that may affect this reaction were considered: quantity of catalyst and reaction time, which were fixed at 10% *w*/*w* (0.4% molar ratio of SO_3_H sites with respect to glycerol) and 10 h, respectively. The authors observed an increase in glycerol conversion and product selectivity with increasing temperature. For the same G:AA molar ratio (1:9), one of the best selectivities toward MAG and TAG, reaching 89% at 40 °C and 57% at 115 °C, respectively, was obtained (Figure 4). However, a 20% decrease in glycerol conversion was observed when accessing SHTC recycling capability, with the major products being MAG and DAG. Even so, upon reuse, it exhibited similar results to those exhibited by carboxylic resins. This would suggest the deactivation of all the sulphonic groups, which was confirmed by solid-state NMR analysis: the formation of sulphonate esters on SHTC’s surface was detected. However, the initial surface state can be easily recovered by acid treatment. 

In the work of Nda-Umar [48], a different behavior in glycerol esterification values was observed upon reutilization of mesoporous sulfonated carbons (OMSC) catalyst derived from palm kernel shell biomass (PKS). Glycerol conversion remained almost constant throughout five catalytic cycles (≈98%). A significant decrease in TAG selectivity was observed, decreasing from 66.5% in the first run to 22.2% in the fifth cycle. Since this is a consecutive reaction, this decrease is relational to an increase in DAG and MAG selectivity, as Calle et al. [47] also observed. Using response surface methodology (RSM), the authors optimized the temperature (126 °C), G:AA molar ratio (1:10.4), catalyst load (0.45 g), and a 3 h reaction time, that managed a glycerol conversion of 97% and a selectivity of 4.9, 27.8 and 66.5% of MAG, DAG and TAG, respectively.

The choice of a temperature of 800 °C under a CO_2_ environment was a result of a previous study by the same group, where through direct, chemical, and template carbonization under CO_2_ environment at 400 and 800 °C, OMSC was produced from PKS biomass and subsequently functionalized with concentrated sulfuric acid [49]. The material carbonized at 800 °C showed the highest selectivity to triacetin (58.9%) with over 97% of glycerol conversion in 3 h reaction time, where the selectivity to MAG and DAG was 5.8 and 32.2%, respectively. This catalyst had a higher TAG selectivity than the well-known Amberlyst-15. These two studies showed that these catalysts have a higher selectivity to TAG and can be reused several times before the selectivity decreases due to the leaching of the active sites of the catalysts.

Rafi and co-workers submitted Karanja seed shells to pyrolysis to prepare biochar for the same purpose, with an optimal carbonization temperature at 400 °C [50]. The authors studied several conditions with a small selectivity toward TAG, including carbonization temperatures. Results concluded that lower carbonization temperatures (300 °C) led to different functional groups, and higher temperatures (500 °C) led to the acidic groups’ loss. Even with the strong acidic sites, glycerol conversion only reached up to 89% for G:AA molar ratio of 1:5, with no significant variation for higher ratios. Similar values (88%) were obtained for reactions with a catalysts weight of 0.2 g. However, compared with the results for a minimum amount of 0.05 g, a 9% increase was obtained with four times the amount of catalyst, increasing conversion from 79% to 88%. No significant variation was observed in glycerol conversion for further increase in catalyst amount. An increase in reaction time up to 4 h gradually increased glycerol conversion. No appreciable enhancement was observed for higher reaction times. An increase in temperature also affected conversion as expected: glycerol conversion increased from 25 to 85% by raising the reaction temperature from 80 to 120 °C. The consistent activity was observed up to five cycles of catalyst reusability without any treatment. No limit was determined for this observation. It would be interesting to see how many more cycles could remain with this activity. The authors compared this catalyst with others reported in the literature, concluding that the lack of treatment/functionalization, lower reaction time, and lower G:AA molar ratio present significant advantages of using this type of biochar.

With one of the highest reusability cycles (up to seven cycles), in the work of Okoye et al. [51], crude bio-derived glycerol was utilized both as a carbon precursor for heterogeneous solid acid catalyst and as a reagent in an acetylation reaction with acetic acid to produce oxygenated fuel additives (DAG and TAG) and MAG. In a single-step method, the heterogeneous solid acid catalyst was synthesized via partial sulphonation and carbonization. Typical sulphonation methods imply the use of temperature; in this work, stirring was the only step applied to add 15 wt.% of concentrated sulfuric acid to glycerol. In one step inside a heated reactor, carbonization, sulphonation, and eventual aromatic polycyclic carbon formation stages were all facilitated by increasing temperature to manage a glycerol-based carbon catalyst (GBCC). With a 49.4% yield, the authors obtained a thermally stable and partially nonporous solid acid catalyst that under the optimal reaction conditions of 110 °C for 3 h with a G:AA molar ratio of 3 with a catalyst loading of 2 wt.%, retrieved an 88% combined yield for DAG and TAG selectivity with a corresponding glycerol conversion of 99%.

In the work of Tao et al. [52] different sulfonated conditions were used to produce sulfonated carbon catalysts of carbonized catkins from willow. Maximal catalyst acidic capacity was obtained with a 90 °C treatment for 5 h. Optimal reaction conditions were set at 120 °C for 2 h with a molar ratio of G:AA of 1:5, with a catalyst amount of 5 wt.% glycerol was almost entirely converted into a mixture of glycerol esters. With a blank test with no sulfonated treatment, the authors proved that conversion of glycerol and selectivity of MAG, DAG, and TAG can be significantly changed with the increase of the acid density of the sulfonated catalyst, a consequence of sulfonated treatment. For the blank experiments, conversion of glycerol (32.0%) and the selectivity to MAG, DAG, and TAG (95.7, 3.6, and 0.7%, respectively) were relatively low. Sulfonated treatment of the catalyst led to higher acidic contents, leading to a higher glycerol conversion (98.4%). Its excellent reusability, proved by its unchanged activity even after six cycles, and its ability to be reused without further treatment showed its potential for glycerol esterification. 

Even with the confirmed potential of all described catalysts (Table 3) and the importance of retrieving TAG derivatives, this selectivity remains limited, with a mixture of MAG, DAG, and TAG being formed, rather than isolated components. Consequently, due to similar boiling points, recovery of each derivative is complicated. Additionally, the production of water shifts equilibrium and often weakens the acidic nature of the catalysts, which is responsible for this selectivity. These prominent reasons and the fact that acetylation with acetic acid is an endothermic reaction, requiring a high energy demand in the formation of TAG, led to the search of better conditions.

**Scheme 4:**
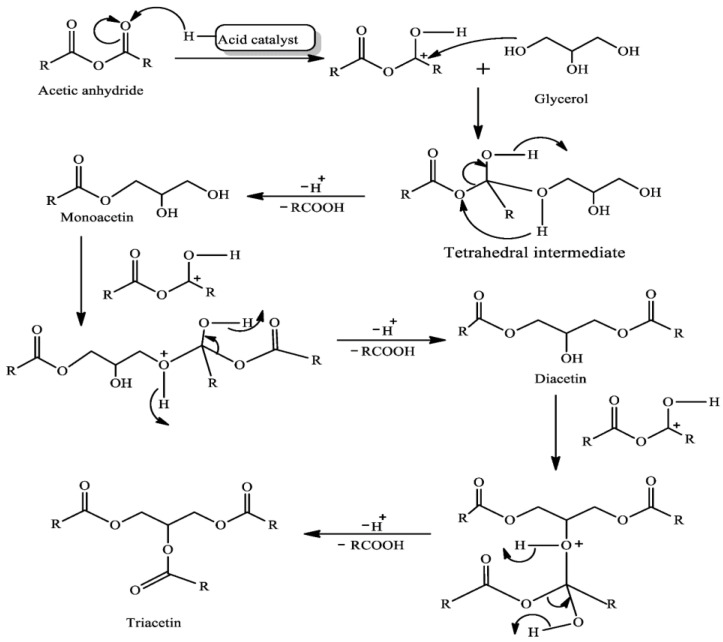
Plausible reaction mechanism for the acetylation of glycerol with acetic anhydride showing the nucleophilic attack leading to the formation of a tetrahedral intermediate and the subsequent formation of MAG, DAG, and TAG (Reproduced with permission of ref. [54]).

Glycerol acetylation with acetic anhydride is an example that obtains in some conditions 100% selectivity toward TAG. The main differences between this reaction and the one that employs acetic acid is its exothermic nature and the byproducts formed. In Scheme 4, a possible mechanism for the acetylation of glycerol with acetic anhydride is presented. Despite its exothermic nature and the fact that the reaction occurs spontaneously, catalyst pore-structure and its acidic nature still strongly influences TAG selectivity. Catalyst pores dimension should be greater than TAG diameter to allow its formation, and this selectivity toward TAG increases with the increase of the density of catalyst surface acid sites. 

**Figure 5:**
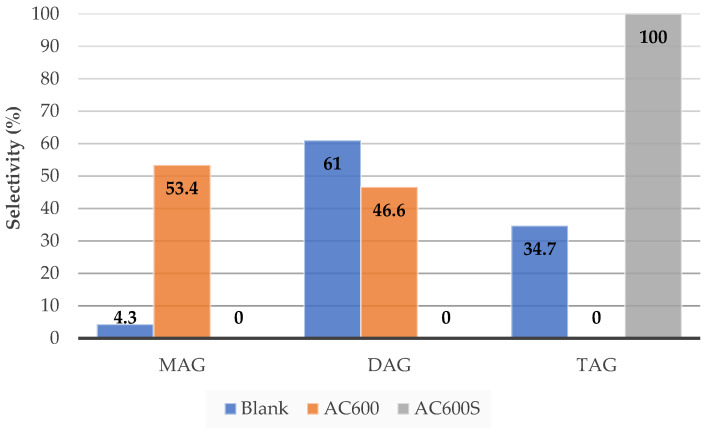
Influence of catalyst on product selectivity at 100 mol % conversion for reaction conditions: ~5 wt% catalyst, T = 100 C; glycerol:Ac_2_O (molar ratio) = 1:5, no stirring, and 50 min reaction time for AC600 and AC600S catalysts and 180 min for the blank experiment [55].

These conclusions were retrieved in the work of Konwar et al. [55] where mesoporous sulfonated carbon catalyst produced by sulfonation of *Pongamia pinnata* de-oiled seed cake (PDOWC) with 4-benzenediazoniumsulfoante were employed in glycerol acetylation with acetic anhydride. The authors obtained 100% of TAG selectivity in 50 min in the presence of one of the developed catalysts (Figure 5). 

**Scheme 5:**
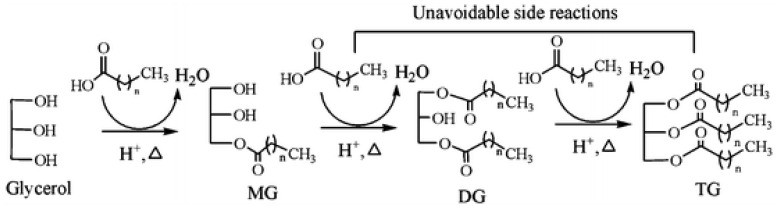
Esterification of glycerol with fatty acids, isomers are not shown (n = 10–18, MG monoglycerides, DG diglycerides, TG triglyceride) (reproduced with permission of ref. [56]).

Using lauric and oleic acids, the same authors [56] studied the esterification of glycerol using the PDOWC catalysts and proposed the mechanism presented in Scheme 5. 

The –SO_3_H containing mesoporous carbon catalyst (ACSO_3_H) was prepared by sulfonation of activated carbon, while the nonporous carbon catalyst (HTCSO_3_H) was prepared by direct hydrothermal treatment with concentrated H_2_SO_4_. Experiments by varying amounts of ACSO_3_H catalyst showed that acid conversion increased as a function of catalyst loading and reached up to 96% at 7.5 wt%. The difference between 5 and 7.5 wt% was very small (<3%) and because of that the 5 wt% catalyst was considered as the optimum amount. These catalysts achieved a selectivity of monoglycerides between 70–80% and fatty acids conversion of 80–95%. The conditions of this experiment were reaction times between 7–24 h, equimolar fatty acid to glycerol ratio and moderate reaction temperatures at 100–125 °C. Moreover, the catalyst was reused and shown to be feasible. Monoglycerides (MG) where the desired product and the conversion and selectivity found to be mainly influenced by acidic properties and pore structure of the catalyst, fatty acid type, and the reaction temperature. With this study, the authors confirmed the catalytic potential of sulfonated carbons as catalyst to produce as well high concentration monoglycerides. 

From the mentioned examples, it can be observed that the catalytic activity of the biochar can be explained based on its properties derived from different characterization methods. The biochar catalysts can be reusable with consistent activity and the acidity of the biochar depended on the carbonization temperature. The conversion and selectivity in glycerol acetylation was also dependent on reaction parameters.

### 2.3. Etherification

**Scheme 6:**
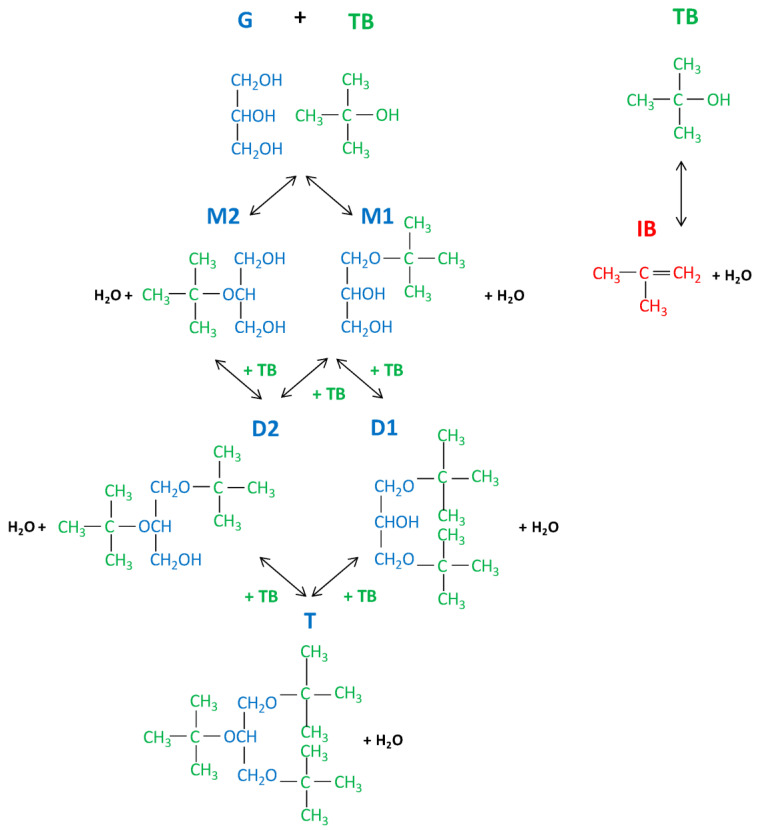
Reaction scheme of glycerol (G) etherification with tert-butyl alcohol (TB) and secondary reaction to isobutene (IB) from TB dehydration (reproduced with permission of ref. [57]).

An important option for glycerol valorization is its conversion into fuel additives, capable of reducing fuel viscosity and density, leading to better engine performance and consequently to less volatile organic compound emissions. The well-known formation of ethers by the dehydration of glycerol—etherification—is a good example of a pathway for such conversion. In the presence of an acid catalyst, alcohols, such as tert-butyl alcohol (TB), are used in the etherification of glycerol. Through a consecutive path (Scheme 6), a nucleophilic attack of glycerol molecules on tert-butyl cations occur, providing a mixture of five tert-butyl ethers, namely monoethers (MTBG), diethers (DTBG), and triether (TTBG). Due to steric hindrance and electrostatic effects, this attack occurs preferentially at primary carbons, and for this reason the formation of monoethers is favored. In fact, the presence of water also favors the formation of monoethers, shifting equilibrium in this direction. However, due to its low solubility in diesel fuel, monoethers are to be avoided, and the generation of higher glycerol ethers (di- and triethers) is preferred. So, the selectivity toward di and triethers should be maximized, to avoid separation procedures and also undesired side reactions, such as, for example, the dehydration of TB to isobutene (IB) and the generation of water that will affect equilibrium [57].

In the work of Gonçalves et al. [58], sulfonated carbon catalysts were prepared through sugar cane bagasse (SCC-S), coconut husk (CHC-S), and coffee ground (CGC-S) (agro-industrial wastes) controlled pyrolysis, for the etherification of glycerol with TB. Functionalization was done with sulphuric acid, which provided a higher number of acidic groups, revealing high activity for glycerol etherification with TBA with a conversion of 81.8%, 61.5%, and 61.5% for SCC-S, CHC-S, and CGC-S, respectively. The catalysts selectively produced MTBG, TTBG, and a small amount of DTBG. The catalyst obtained from sugar cane bagasse showed good catalytic activity in glycerol, reaching conversion as high as 80% in a short reaction time (4 h) and a selectivity of 21.3%. One of the lowest yields (14%) of DTBG + TTBG was achieved by the CGC-S catalyst after 4 h of reaction. The CHC-S catalyst yielded 21.1% of DTBG + TTBG, but the concentration of mono-ether was lower than 40%. In this study a slight catalyst deactivation was observed after the fourth cycle in which the conversion decreased to 86%. Different deactivation factors, such as leaching and blocking of sites, could be responsible for the vulnerability of the active sites. The sulphur content slightly decreased from 1.7% to 1.6%, corresponding to a reduction of approximately 6% after the fourth cycle. Additionally, this study confirmed that the presence of the sulfonic groups in a flexible and hydrophilic structure was probably the key factor for the improved catalytic performance of these carbons.

In another study, M. Gonçalves and co-workers [59] focus on treating the environmentally friendly black carbons derived from coffee grounds with sulfuric acid (BCC-S) or fuming sulfuric acid (BCC-SF). The catalyst treated with fuming sulfuric acid was performed under reflux at 70 °C with different times, 2, 5, and 10 h, and then washed. These materials were then utilized as catalysts in glycerol etherification reactions with TBA under inert atmosphere and 5% of catalyst (wt.% of glycerol), and a molar ratio of 4:1. The BCC-SF catalyst showed the highest amount of sulfur groups, approximately 8%. However, these two catalysts demonstrated high catalytic activity in the etherification of glycerol with TB, the activity being higher for the BCC-SF. In the case of the BCC-SF, the yields were approximately 40 and 20% for the mono-tert-butyl glycerol (MTBG) and di-tert-butyl glycerol + tri-tert-butyl glycerol (DTBG + TTBG), respectively, after 2 h of reaction. This is explained by the number of acid sites, principally the high amount of sulphur groups. Furthermore, the black carbons catalysts show a high stability of the active groups and can be reused in consecutive reactions [59].

This group also used carbonaceous wastes, such as glycerine (G), waste coffee grounds (WC), and polyethylene terephthalate (PET), as precursors to obtain carbon catalysts to transform crude glycerol into value-added products via glycerol etherification. This experiment showed promising results for glycerol etherification with TBA, reaching approximately 80% conversion and a high selectivity for the MTBG species (3-tert-butoxy-1,2-propanediol). The catalyst obtained from PET (S-CPET) waste demonstrate one of the highest glycerol conversions and selectivity for MTBG and can reach a dynamic equilibrium at 4 h of reaction. This type of catalyst showed the highest amount of sulphur groups, which explains these results. The catalyst with WC (S-CWC) waste was the other material that demonstrates having one of the highest glycerol conversions and selectivity for MTBG and, similar to the other catalyst, reaches dynamic equilibrium at 4 h of reaction. These two types of catalysts were the best in converting glycerol and in having the higher selectivity, probably due to their high acidity. In addition, the absence of a porous structure (micro and mesoporous) could explain the high selectivity for MTBG. Due to the prepared carbons having a low surface area, the etherification reaction occurs over the acid’s sites, and the formed products, such as MTBG, are desorbed into the reaction medium. Additionally, these carbon catalysts can be reused in several reaction cycles. These studies demonstrated environmentally friendly acid black carbon catalysts produced from abundant waste, such as coffee grounds. This catalyst proved to have good activity in the etherification reaction, and the catalytic surface is very stable [60].

The development of a sulfonated carbon catalyst with olive stones and the sulfonic acid groups incorporated in the catalyst by sulfuric acid treatment was carried out by Estevez and co-worker’s [61] at different sulfonation times (0.5, 2 and 5 h) and temperatures (100 and 150 °C). Either conventional heating or microwave irradiation was used, and regardless of the sulfonation conditions, all the solids exhibited similar particle sizes. Thus, using microwave irradiation in the preparation of the sulfonated carbons was shown to be positive. A total of 30 min of sulfonation under microwave irradiation led to a ~3.5% of sulphur, while it took 2 h for conventional heating to attain the same percentage. Then, in microwave-assisted etherification of glycerol with TB at autogenous pressure, 75 °C and 15 min of reaction is comparable with other sulfonated carbons but using higher temperatures and reactions times. The sulfonated carbons exhibited high stability, and after the fifth use of the catalyst, the yield value remained practically constant. 

In Table 4 a comparison is provided for all the above-mentioned catalysts and their best performance according to the optimal reaction conditions. Even though the advantage of using such types of biobased catalysts is still important, the range in glycerol conversion is much lower than in the other reported cases for transesterification and esterification reactions. Another important fact is that selectivity toward MTBG is much higher, and lower selectivity toward the DTBG and TTBG represents a problem according to the main goal that is to obtain the so-called high ethers.

Using a commercial catalyst (Amberlyst) as a comparison, Frusteri et al. [62] investigated the hydrothermal carbonization process (HTC) under moderate conditions (200 °C) to obtain amorphous carbon microspheres (200–300 nm in size) and spheres of 1–2 μm, using glucose (as a carbon precursor) in aqueous solution. Functionalized with inorganic acids and by impregnating a superacid ionomer (Hyflon) dissolved in alcohol solution, these catalysts proved to be good alternatives for carrying out the etherification of glycerol with isobutylene. Experiments were conducted at 70 °C, under autogenous pressure (2–11 bar), with a catalyst amount of 7.5 wt.% and a glycerol:isobutene molar ratio of 1:4.

**Figure 6:**
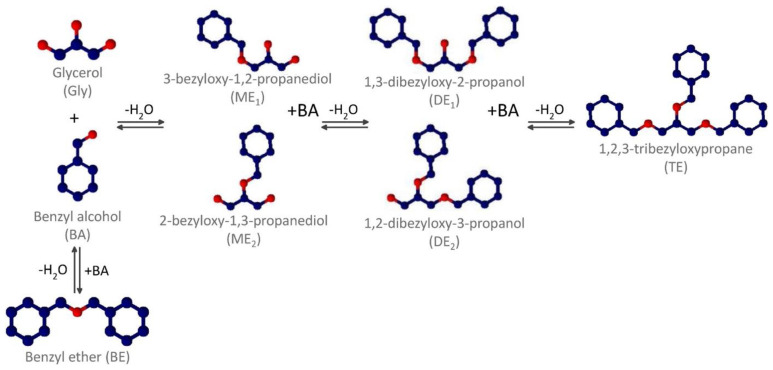
Etherification reaction of glycerol (Gly) with benzyl alcohol (BA) (reproduced with permission of ref. [63]).

The purpose of the work was to find alternatives to Amberlyst catalyst that with isobutylene tends to oligomerize, forming diisobutene (DIB), competing with etherification reaction, and consequently reducing the conversion of glycerol. The problem of this and other side-products, which must be removed from the ether mixture, is the deposition into engines, when used as additive for diesel reformulation. Despite their lower acidity in comparison with Amberlyst catalyst, the authors found out that the studied catalysts showed an excellent catalytic activity in terms of glycerol conversion, while the formation of oligomers was strongly inhibited. With the purpose of searching for new derivatives of benzyl alcohol (BA) to be employed as solvent in hair dyes to resolve dermal irritation, this alcohol has been used in the etherification of glycerol. In Figure 6, a possible mechanism is presented for the etherification of glycerol with BA. Two monoethers can be formed: 3-benzyloxy-1,2-propanediol (ME1) and 2-benzyloxy-1,3-propanediol (ME2); two diethers: 1,2-dibenzyloxy-3-propanol (DE1) and 1,3-dibenzyloxy-2-propanol (DE2); and a triether: 1,2,3-tribenzyloxypropane (TE). Side reactions can also occur forming benzyl ether (BE).

Presenting the same problem as the esterification process, the formation of water during the reaction can shift equilibrium toward the reactants, decreasing the formation of diethers (DE) and triethers (TE). The solution is to continuously remove water from the process, turning it to an irreversible process.

**Figure 7:**
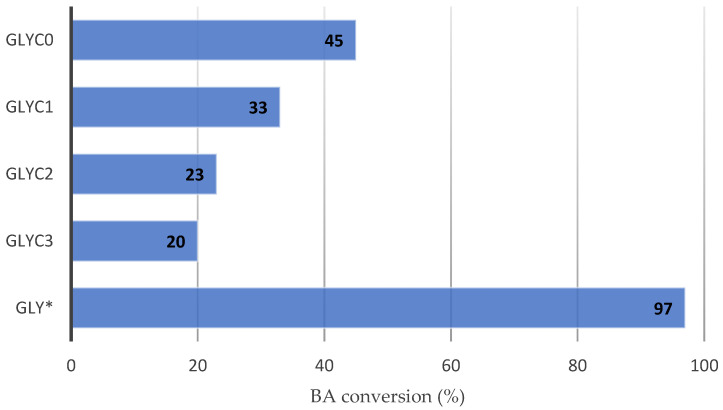
Results of etherification of GlyC with BA. Temperature of 120 °C, ratio Gly: BA = 3:1 and 10 wt.% of catalyst. (GlyC0: 12.36% moisture, GlyC1: 8.17% moisture, GlyC2: 5.78% moisture, GlyC3: 2.36% moisture and Gly* Glycerol refined) [63].

Chiosso and co-workers [63] developed a sustainable, inexpensive carbonaceous system (Ccs), obtained via a synthetic method with low energetic cost in only 24 h. They functionalized it with –SO_3_H groups and utilized them as catalysts in the etherification of glycerol (Gly) with benzyl alcohol (BA). They compared crude (GlyC) and refined glycerol (Gly) (Figure 7). The catalyst performed the best (97% conversion) with commercial glycerol (Gly) at 120 °C, with a Gly:BA ratio of 3:1 and 10 wt.% of Ccs, after 360 min of reaction. Due to its acidity, it could be possible to lower the reaction temperature and the catalyst concentration (2.5 wt.%) without observing a significant loss in BA conversion. In the case of GlyC samples, the authors improved the BA conversion in samples with lower water content. However, it was observed that the Ccs catalyst was selective toward ME1, ME2, and DE1. In addition, the formation of water and BE was also observed.

Reusability was only studied in three catalytic cycles at 120 °C, Gly:BA molar ratio = 3:1 and 10 wt.% of catalyst. Results showed that conversion and selectivity were only maintained within the first and second use. The principal cause of activity loss was leaching.

Due to its inexpensive nature, activity, selectivity, and reusability, the authors classified this material as a promising alternative for crude glycerol conversion into value-added chemicals.

Common to all the described examples, the most active catalysts for etherification of glycerol must be strongly acidic. It will be important to develop more studies regarding the effect of the porosity of the supports to understand their effect on selectivity and yield. In addition, the oxygen-rich biomass parent molecule, glycerol, can be used for fuel-blending [64].

### 2.4. Acetalization/Ketalization

Acetalization/ketalization is one of the most useful methods for the protection of al-dehydes/ketones. Acetalization reaction to produce cyclic acetals or ketals is favored by the presence of Bronsted acid catalysts [65]. Normally, acetalization of glycerol with either aldehydes or ketones is carried out using homogeneous catalysts and mineral acids, such as HCl, H_2_SO_4_, and HF, which are corrosive [66,67]. However, lately several studies have been published describing many suitable heterogeneous catalysts, such as Amberlyst resins [68], mixed oxides [69], and functionalized activated carbons, giving focus to more sustainable and environmentally friendly processes [65,70].

In the work of Gonçalves and co-workers [71] highly selective acetalization of glycerol with acetone was performed with acidic carbon-based catalysts obtained from biodiesel waste to produce solketal. The production of the acid carbon-based catalyst (GC) was made by hydrothermal carbonization of a mixture of glycerin and sulfuric acid at different mass ratios under 150 °C for 24 h and with different mass ratios. After carbonization, the catalysts were repeatedly washed with distilled water until neutral pH was achieved and followed by oven-drying at 120 °C for 24 h. The authors observed high catalytic activity, reaching up to 82% of glycerol conversion with a selectivity toward solketal of almost 90%.

**Figure 8:**
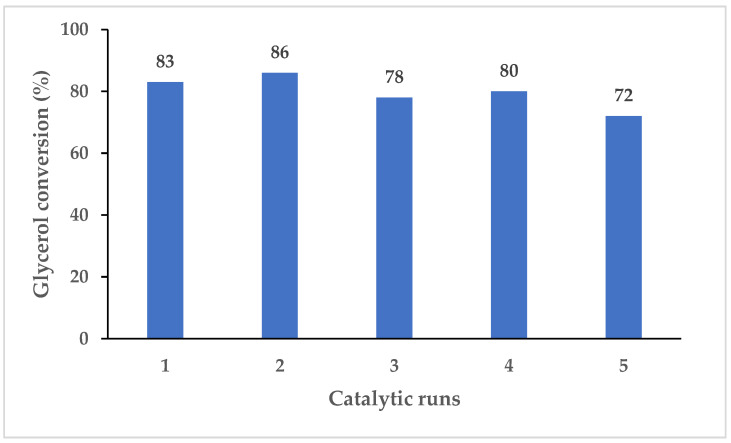
Glycerol acetalization in successive catalytic runs. Reaction conditions: Glycerol:acetone molar ratio 1:4, 3% of catalyst and 120 min of reaction time.

Regarding its reusability, the authors observed that removing the catalyst after each catalytic run would stop the reaction progress. This is an indication that there is no appreciable leaching of any active groups present on the catalyst surface. In fact, the authors consider that only a slight difference in the glycerol conversion was observed, being the overall catalytic activity of the catalysts roughly maintained through five experiments as observed in Figure 8.

**Figure 9:**
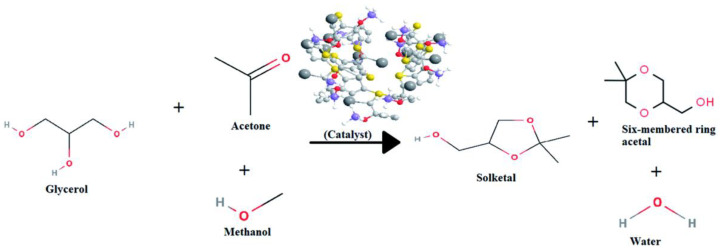
Glycerol ketalization with acetone and excess of methanol [72].

Lower conversions were obtained in the work of J. Kaur [72] and co-workers, where value-added carbon produced from *Zea mays* L. cob was activated by NOH (Figure 9) and used to produce solketal. The optimum conditions achieved with a molar ratio of 1:8:8 (glycerol:acetone:methanol) in 1 h with 5 wt% (w.r.t. glycerol) of catalyst at 50 °C. These optimum conditions provide a maximum glycerol conversion of 72% and the reusability was only tested until the 3rd consecutive reaction. At the end of this test a little deformation in the structure of the catalyst was observed, although the percentage of carbon remains almost the same (72%) as the original catalyst. This deformation seems to be linked to the reduction of the conversion efficiency from glycerol to solketal, which was less than 5%. 

With higher conversion values at approximately 90% (0.6 wt% catalyst, 1:10 glycerol:2- propanone after 2 h), Fabiane and co-workers [73] studied the solvent-free glycerol ketalization for the production of 2-propane solketal, using amphiphilic carbon catalyst produced by bio-oil sulphonation. The authors state that using this type of catalyst synthesis, amorphous carbon with high concentration of hydrophilic oxygen and sulfonic surface groups with strong acid sites could be prepared. They also reported that no significant loss in catalytic activity was observed after four consecutive reactions. 

## 3. Conclusions

The use of biochar as catalysts for transesterification reaction is a growing topic in the last couple of years. The main advantages of biochar are based in their relatively high surface area, tailored textural characteristics, and low cost. They are sourced from several origins and have showed excellent results for transesterification reactions, such as conversions up to 100%, good reusability conditions with small deactivation percentages that could be reversed through reactivation with small additions prior to reuse.

In the case of esterification, examples where dual-purpose reagents are used can be a good approach. The work of Okoye et al. [51] can be an interesting starting point to promote acetylation reaction with acetic acid to produce oxygenated fuel additives (DAG and TAG) and MAG. The most important difficulty pointed out by the researchers is the limited selectivity toward MAG, DAG and TAG formation. Due to their similar boiling points the recovery of each derivates can be complicated.

For etherification reaction a sustainable, inexpensive carbonaceous system (Ccs), appears to be a good choice due to the possibility of a functionalization method with low energetic cost in only 24 h, despite its lower conversion yields. However, the selectivity toward MTBG is much higher and may represent a problem to address. 

Thus, the field of biochar catalysts for glycerol valorization can be considered relatively young, and it offers the possibility to explore a variety of interesting catalytic materials. In addition, the hypothesis of using a waste that would probably be discarded or burned is certainly an advantage to consider.

## Figures and Tables

**Table 1:** Comparison of CCR-500 catalyst with two reported catalysts representing low and high cost catalysts of glycerol (G) with dimethyl carbonate (DMC) to glycerol carbonate (GC).

Catalyst	Feedstock	Glycerol Conversion Yield (%)	Reuse Cycles	Glycerol Conversion Yield after the Last Reuse (%)	Catalyst-to-Feed Ratio	Ref.
CCR-500	Corncob	98	4	78	0.12	[39]
K-zeolite	KOH, coal fly ash	100	4	94	n.a.	[40]
NiFe_2_O_4_@(CaO-La_2_O_3_)	Ni(NO_3_)_2_, Fe(NO_3_)_3_, CaCl_2_ and La(NO_3_)_3_	99	6	95	n.a.	[41]

**Table 2:** Comparison of biochar catalysts performance for the transesterification of glycerol (G) with dimethyl carbonate (DMC) to glycerol carbonate (GC).

Catalyst Feedstock	Catalyst Loading (wt.%)	G:DMC Molar Ratio	T (°C)	Time (min)	G Conversion (%)	GC Yield (%)	Catalyst-to-Feed Ratio	Ref.
*musa acuminata* peel ash (MAPA)	6	1:2	75	15	99	99.5	0.59	[34]
Fishmeal (FMB)	2	1:2	85	60	100	99.5	0.02	[36]
Cow dung (CDB)	3	1:1	85	90	100	97.1	0.03	[38]
Fish scale (FSB)	2	1:2	85	60	100	99.6	0.02	
Corncob residue (CCR-500)	3	1:3	80	90	98	94.1	0.12	[39]

**Table 3:** Selectivity of all above described catalysts according to the optimal carbonization temperature and reaction conditions.

Catalyst Feedstock	Carbonization T (°C)	T (°C)	Time (h)	G:AA Molar Ratio	Glycerol Conversion (%)	MAG (%)	DAG (%)	TAG (%)	Ref.
D-glucose	195	115	10	1:9	98	5	38	57	[47]
Palm kernel shell biomass	800	126	3	1:10.4	97	4.9	27.8	66.5	[48]
	800	120	3	1:6	97	5.8	32.2	58.9	[49]
Karanja seed shells	400	120	4	1:5	89	56	40	4	[50]
Crude glycerol	-	110	3	3	99	12		88	[51]
Sucrose	400	180	4	1:9	>99	-	-	50	[53]
Catkins from willow	90	120	2	1:5	98.4	32.8	54.5	12.7	[52]

**Table 4:** Selectivity toward MTBG, DTBG, and TTBG of all above described catalysts according to the optimal reaction conditions.

Catalyst Feedstock	T (°C)	Time (h)	G:Solvent Molar Ratio	Glycerol Conversion (%)	MTBG (%)	DTBG (%)	TTBG (%)	Ref.
Sugar cane bagasse	120	4	1:4	80	-	21.3		[58]
Waste coffee ground	120	4	1:4	~70	~42 ^a^	~20 ^a^		[59]
Waste coffee ground	120	8	1:4	>65	>80	>30	~10	[60]
Polyethylene terephthalate (PET)				80	70	<30	<5	
Crude glycerine				79	68	25	7	
Olive stones	75	15 min	1:4	84	74	26		[61]

^a^ Values for product yield.

## Data Availability

Not applicable.

## Sample Availabilitys

Not available.
